# Negatively Regulated by miR-29c-3p, MTFR1 Promotes the Progression and Glycolysis in Lung Adenocarcinoma *via* the AMPK/mTOR Signalling Pathway

**DOI:** 10.3389/fcell.2021.771824

**Published:** 2021-12-01

**Authors:** Yongmeng Li, Yanfei Liu, Kai Jin, Rui Dong, Cun Gao, Libo Si, Zitong Feng, Huiying Zhang, Hui Tian

**Affiliations:** ^1^ Department of Thoracic Surgery, Qilu Hospital, Cheeloo College of Medicine, Shandong University, Jinan, China; ^2^ Department of Anesthesiology, Qilu Children’s Hospital of Shandong University, Jinan, China

**Keywords:** lung cancer, microRNA, mitochondrial fission regulator 1, Warburg effect, proliferation, invasion, migration

## Abstract

**Background:** Lung adenocarcinoma (LUAD) is the major form of lung cancer that presents a major peril to public health. Owing to the high rates of morbidity, mortality and chemoresistance, it is necessary to develop more effective therapeutic targets of LUAD. Mitochondrial fission regulator 1 (MTFR1) affects the occurrence and development of some diseases by regulating mitochondrial dynamics and is dysregulated in LUAD. However, the functions and molecular mechanisms of MTFR1 in LUAD have not been investigated.

**Methods:** Immunohistochemical (IHC) analysis, real-time quantitative polymerase chain reaction (RT-qPCR), bioinformatic analysis and western blot (WB) were performed to assess the expression of MTFR1 at both protein and mRNA levels. The biological functions of MTFR1 in LUAD cells were assessed based on various *in vivo* and *in vitro* experiments. The dual-luciferase reporter assay and some rescue experiments were performed to evaluate the underlying mechanism of MTFR1 in LUAD.

**Results:** MTFR1 was upregulated in LUAD cells and tissues and correlated with dismal clinicopathologic features and a worse prognosis of patients with LUAD. Functionally, MTFR1 overexpression stimulated the proliferation, invasion, migration and glycolytic capacity and impeded the apoptosis of LUAD cells; however, opposite results were obtained when MTFR1 expression was knocked down. MTFR1, which was directly targeted by miR-29c-3p, may exert its biological functions through the AMPK/mTOR signalling pathway.

**Conclusion:** MTFR1 promotes the progression of LUAD. Therefore, targeting MTFR1 can offer an effective therapeutic strategy for LUAD treatment.

## Introduction

Lung cancer is a major contributor to cancer-associated mortality worldwide, and non-small cell lung cancer (NSCLC) is the major subtype constituting approximately 85% of all lung cancer cases (Chen et al., 2016; Sung et al., 2021). LUAD has been recognised as the most common NSCLC subtype which has high morbidity and fatality rates (Herbst et al., 2018). Although patients with LUAD have benefited significantly from the development of targeted therapy and immunotherapy in recent years, their clinical prognosis remains unsatisfactory. Therefore, it is necessary to discover novel therapeutic targets and biomarkers for LUAD.

Studies have revealed that abnormal metabolism is a typical characteristic of cancer cells and a key contributor to cancer progression (Feng and Levine, 2010; Hanahan and Weinberg, 2011). Mitochondria are the main energy-generating organelles, which are responsible for cellular metabolism in eukaryotic cells (McBride et al., 2006). Mitochondria are in constant dynamic equilibrium between fission and fusion to adapt to functional changes (Chan, 2006; Knott et al., 2008; Chan, 2012). The disruption of this balance may contribute to the occurrence of different diseases, including different types of cancers (Lin and Beal, 2006; Guo et al., 2020). Recent studies have indicated that dysfunctional mitochondrial fission is involved in the progression of some malignancies, including glioblastoma, liver cancer and gastric cancer (Hernandez-Alvarez and Zorzano, 2021; Lenzi et al., 2021; Zhu et al., 2021). Furthermore, some studies have indicated that mitochondrial dynamics have an imperative function in the chemoresistance of cancer cells (Kong et al., 2015; Cai et al., 2016; Huang et al., 2018). Mitochondrial fission regulator 1 (MTFR1), previously called CHPPR or FAM54A2, is a mitochondrial protein with a polyproline-rich region (Tonachini et al., 2004). Some studies have indicated that MTFR1 can promote mitochondrial fission and regulate cellular respiration (Tonachini et al., 2004; Monticone et al., 2010). In addition, MTFR1 has a vital function in oral squamous cell carcinoma, acute myocardial infarction (AMI) and head and neck squamous cell carcinoma (HNSCC) (Wang et al., 2015; Reddy et al., 2019; Huang et al., 2020). Moreover, bioinformatic analyses in a study have indicated that MTFR1 is dysregulated in LUAD (Sanada et al., 2019). However, the function and mechanism of action of MTFR1 in LUAD remain investigated.

MicroRNAs (miRNAs), which are small non-coding RNAs spanning approximately 21 nucleotides, usually impede gene expression by binding to the 3′ untranslated region (3′-UTR) of their target mRNAs (Bartel, 2004). A study has indicated that miRNAs are involved in the regulation of most mammalian mRNAs (Friedman et al., 2009). Therefore, it does not come as a surprise that the dysregulation of miRNA expression may cause a multitude of diseases including cancers (Jiang et al., 2009; Li et al., 2021). miR-29c-3p suppresses the progression of various cancer types, including intrahepatic cholangiocarcinoma, cervical cancer, ovarian cancer and oesophageal cancer (Wang H. et al., 2020; Feng et al., 2020; Hozaka et al., 2021; Zou et al., 2021). However, to date, the expression level and function of miR-29c-3p in LUAD have yet to be investigated.

In this study, we aimed to assess the expression level, biological role and underlying molecular mechanism of MTFR1 in LUAD. We reported for the first time that MTFR1 is overexpressed in LUAD cells and tissues. Furthermore, MTFR1 stimulated the progression of LUAD both *in vivo* and *in vitro*. We propose that MTFR1 is a direct target of miR-29c-3p and may exert its biological effects *via* the AMPK/mTOR signalling pathway. Therefore, this study offers novel insights into the role of MTFR1 as an effective therapeutic target for LUAD treatment.

## Methods

### Data Processing

Gene expression data were downloaded from The Cancer Genome Atlas (TCGA, https://tcga-data.nci.nih.gov/tcga/), Genome–Tissue Expression (GTEx, https://www.ncbi.nlm.nih.gov/geo/) and Gene Expression Omnibus (GEO, https://www.ncbi.nlm.nih.gov/geo/) databases. The target miRNAs were predicted using the following bioinformatics software: miRbase (www.mirbase.org), starBase (http://starbase.sysu.edu.cn), miRWalk (www.umm.heidelberg.de/apps/zmf/mirwalk) and miRDB (http://mirdb.org/miRDB).

### Collection of Clinical Tissue Specimens

A total of 85 LUAD tissues and matched paracancerous normal tissues of the lung were obtained from our tissue bank to establish the tissue microarray. All tissue samples were extracted through surgical resection at the Department of Thoracic Surgery, Qilu Hospital of Shandong University from 2004 to 2014. In addition, 20 paired fresh LUAD tissues and corresponding paracancerous tissues were obtained immediately after surgical resection in patients with LUAD admitted to our department. This study was reviewed and approved by the Medical Ethics Committee of Qilu Hospital of Shandong University (Approval No. KYLL-2016-097). The patients included in this study or their family members were requested to sign an informed consent document.

### Cell Culture and Treatment

A total of five LUAD cell lines (H1299, PC9, A549, H1975, and H157), human embryonic kidney 293 (HEK293) cells and human bronchial epithelial (HBE) cells were provided by the Shanghai Academy of Science (Shanghai, China). The cell lines were cultured in RPMI-1640 medium supplemented with 10% foetal bovine serum (FBS; Gibco, NY, United States). The cells were incubated in a moistened environment with 5% CO_2_ at 37°C. The sh-NC, sh-MTFR1, oe-NC and oe-MTFR1 lentiviruses (Jikai Co., Shanghai, China) were transduced into the corresponding cells. The sh-RNA sequences are listed in Supplementary Table S1. The cells that were stably transduced were selected using puromycin (2 μg/ml) for 7 days after transduction. Mimics-miR-29c-3p, inhibitor-miR-29c-3p and their corresponding negative controls (NCs) were obtained from GenePharma Co., Ltd. (Shanghai, China). The oligonucleotide sequences were as follows: mimics-miR-29c-3p (5′-UAG​CAC​CAU​UUG​AAA​UCG​GUU​A-3′), mimics-NC (5′-UUC​UCC​GAA​CGU​GUC​ACG​UTT-3′), inhibitor-miR-29c-3p (5′-UAA​CCG​AUU​UCA​AAU​GGU​GCU​A-3′) and inhibitor-NC (5′-CAG​UAC​UUU​UGU​GUA​GUA​CAA-3′). The cells were transfected with miRNAs using the jetPRIME transfection reagent (Polyplus-transfection, Illkirch, France) according to the manufacturer’s protocol.

### Western Blot

Proteins extracted using the radioimmunoprecipitation assay (RIPA) reagent were quantified using a BCA kit (Biyuntian, China). Subsequently, the protein lysates were separated using 6% or 10% sodium dodecyl sulfate-polyacrylamide gel electrophoresis (SDS-PAGE) gels and transferred onto polyvinylidene difluoride (PVDF) membranes. Furthermore, 5% non-fat milk was used to block the membranes at ambient temperature for 1 h. The membranes were subsequently washed with TBST (thrice for 10 min each) and were incubated overnight with primary antibodies at 4°C, followed by incubation with a secondary antibody at ambient temperature for 1 h. Eventually, bands were visualised using an enhanced chemiluminescence (ECL) system. β-tubulin was used as the internal control. The following antibodies were used in this study: anti-N-cadherin (ab18203, Abcam), anti-MTFR1 (ab198192, Abcam), anti-Vimentin (ab92547, Abcam), anti-p-mTOR (ab109268, Abcam), anti-E-cadherin (ab40772, Abcam), anti-mTOR (ab32028, Abcam), anti-Snail (ab216347, Abcam), anti-p-AMPK (ab133448, Abcam), anti-Slug (ab27568, Abcam), anti-Ki-67 (ab16667, Abcam), anti-AMPK (ab32047, Abcam), anti-PARP (ab32064, Abcam), anti-BAX (32503, Abcam), anti-BCL-2 (ab32124, Abcam) and anti-β-Tubulin (ab18207, Abcam).

### RNA Isolation and Real-Time Quantitative Polymerase Chain Reaction

Extraction of Total RNA was extracted using the RNAfast200 kit (Fastagen, Shanghai, China). Complementary DNA (cDNA) was synthesised using a reverse transcription kit (Toyobo, Osaka, Japan) according to the manufacturer’s instructions. RT-qPCR was then executed in Bio-Rad IQ 5 system (Bio-Rad) using the SYBR Green Supermix (Toyobo, Osaka, Japan). The primers used include the following: *MTFR1* (F, 5′-TGC​AAC​AGA​ATG​GAG​TCC​CA-3′ and R, 5′-AAG​GGG​TGG​CCT​TGA​TCT​GA-3′); *GAPDH* (F, 5′-GCA​CCG​TCA​AGG​CTG​AGA​AC-3′ and R, 5′-TGG​TGA​AGA​CGC​CAG​TGG​A-3′); miR-29c-3p (F, 5′-CTC​CTC​CTT​TTA​GCA​CCA​TTT​G-3′ and R, 5′-TAT​GCT​TGT​TCT​CGT​CTC​TGT​GTC-3′) and *U6* (F, 5′-CAG​CAC​ATA​TAC​TAA​AAT​TGG​AAC​G-3′ and R, 5′-ACG​AAT​TTG​CGT​GTC​ATC​C-3′). *U6* and *GAPDH* were used as internal references, and all the aforementioned primers were obtained from GenePharma Co., Ltd. (Shanghai, China). The experiment was repeated thrice, and data were analysed using the 2^−△△Ct^ method.

### Haematoxylin–Eosin Staining and Immunohistochemical Analysis

First, the formalin-fixed and paraffin-embedded tissues samples were split into smaller sections of 4 µm. For HE staining, each section was dewaxed, rehydrated and stained with HE, and images were captured using an inverted microscope. With regards to IHC, each section was dewaxed, rehydrated and incubated with primary antibodies overnight at 4°C, followed by incubation with a secondary antibody at ambient temperature for 1 h. Subsequently, the sections were viewed, and an inverted microscope was used to capture images. Lastly, histochemical scoring (H-score) was used to assess the results of IHC analysis as previously described (Xie et al., 2014). The following antibodies were used: anti-MTFR1 (ab198192, Abcam) and anti-Ki-67 (ab16667, Abcam).

### Cell Counting Kit-8 Cell Proliferation Assay

The transduced cells were seeded into 96-well plates (2,000 cells/well) and cultured for 24, 48, 72, and 96 h, sequentially. Subsequently, 10 µL of CCK-8 solution (5 mg/ml; Solarbio, China) was added to each well. After incubating the cells for 2 h in dark, absorbance was recorded at 450 nm using a microplate reader. The experiment was performed in triplicates.

### EdU Staining

The transduced cells were seeded into 96-well plates (5 × 10^3^/well) and incubated with complete RPMI-1640 medium containing 5-ethynyl-2-′deoxyuridine (EdU; 1000:1) for 2 h. EdU staining was performed using an EdU kit (RiboBio, Guangzhou, China) according to the manufacturer’s protocol. Lastly, the stained cells were photographed using a fluorescence microscope. The experiment was performed in triplicates.

### Colony Formation Assay

The transduced cells were seeded in 6-well plates (1,000 cells/well) and incubated for 15 days. Then the cells were fixed with 4% paraformaldehyde for 30 min and stained with 0.1% crystal violet for 15 min. Subsequently, the cells were washed thrice with phosphate-buffered saline (PBS). Lastly, a digital camera was used to capture images. The experiment was performed in triplicates.

### Analysis of Cell Cycle and Apoptosis

The cell cycle and apoptosis were assessed using a flow cytometer (BD Biosciences, United States). A propidium iodide (PI) staining kit (Yeasen, Shanghai, China) was used to analyse the cell cycle, whereas the Annexin V-APC Apoptosis Detection Kit (eBioscience, Thermo Fisher) was used to assess cell apoptosis according to the manufacturer’s protocol. The experiments were performed in triplicates.

### Wound-Healing Assay

The transduced cells were seeded in 6-well plates until they attained confluency. Subsequently, a graze in the monolayer was created using the tip of a 200-μL pipette. After three PBS washes, the medium was replaced with serum-free media (SFM). Subsequently, the width of the wound was visualised and assessed under a microscope at 0 and 24 h. The experiment was performed in triplicates.

### Transwell Assay

Cell suspensions (5 ×  10^4^ cells/200 uL of SFM) were added to the upper Transwell chamber with or without Matrigel, whereas 600 uL of a medium supplemented with 20% FBS was added to the lower Transwell chamber. After 24 h, the cells in the upper chamber were removed, and those that entered or moved to the lower chamber were fixed using 75% ice-cold alcohol for 30 min. Subsequently, the cells were stained with 0.1% crystal violet for 20 min, and an inverted microscope was used to capture images. The experiment was performed in triplicates.

### Measurement of Glucose, Lactate and Extracellular Acidification Rate

The transduced cells were seeded in 6-well plates (1 × 10^5^ cells/well) and incubated for 48 h. Subsequently, the culture medium was harvested, and the levels of glucose and lactate were measured using the CheKine™ Glucose Assay Kit (Abbkine, Wuhan, Hubei, China) and CheKine™ Lactate Assay Kit (Abbkine, Wuhan, Hubei, China), respectively, according to the manufacturer’s protocol. For ECAR measurement, cells were seeded into a Seahorse XF 96 cell culture micro-plate and incubated overnight (1 × 10^4^/well; Agilent Technologies). The next day, a Seahorse XFe96 Analyzer (Agilent Technologies) was used to measure the ECAR with a Seahorse XF Glycolysis Stress Test Kit (Agilent Technologies) following the manufacturer’s protocols. Data were analysed by the Seahorse XF 96 Wave software. The above experiments were performed in triplicates.

### Dual-Luciferase Reporter Assay

HEK-293T cells were seeded into 24-well plates (5 × 10^4^ cells/well). After incubating the cells for 24 h, they were transiently co-transfected with mimics-NC or mimics-miR-29c-3p together with the mutated (MUT) 3′-UTR or wild-type (WT) *MTFR1* mRNA. After 48 h, the luciferase activity was assessed using a dual-luciferase assay kit (Promega). The experiment was replicated in triplicates.

### *In Vivo* Experiments

We procured 4-week-old BALB/c nude mice from GemPharmatech Co., Ltd. (Nanjing, China). To establish a xenograft tumour model, 20 nude mice were categorised randomly into four cohorts. Subsequently, 5 × 10^6^ transduced A549 cells (LV-sh-MTFR1 and LV-sh-NC) or PC9 cells (LV-oe-MTFR1 and LV-oe-NC) were hypodermically implanted into the right armpit area of each mouse. The tumour size was measured every 4 days. The tumour volumes were computed using the following formula: V = (length × width^2^)/2. The nude mice were sacrificed on the 28th day, and all tumours were collected and weighed. To construct an experimental model for lung metastasis, 20 nude mice were randomly divided into four cohorts, and 2 × 10^6^ transduced cells were introduced into the mice *via* an injection in the tail vein. The mice were euthanised after 2 months, and their lungs were obtained. All specimens were further stained with IHC or HE. The animal experiments were approved by the Shandong University Animal Research Ethics Committee under a project license (SCXK Lu 20090001).

### Statistical Analysis

Data were expressed as the mean ± standard deviation (SD). Data were analysed using the GraphPad Prism (GraphPad version 8) and R (version 4.0.3) software. The Student’s *t*-test (two-sided), chi-square test, Pearson’s correlation analysis and Kaplan–Meier (KM) survival analysis were performed as indicated. Multiple groups were compared for identifying statistically significant differences using one-way analysis of variance (ANOVA). The observed differences were considered statistically significant at *p* < 0.05.

## Results

### MTFR1 was Overexpressed in LUAD Cells and Tissues and Associated With a Poor Prognosis

**FIGURE 1:**
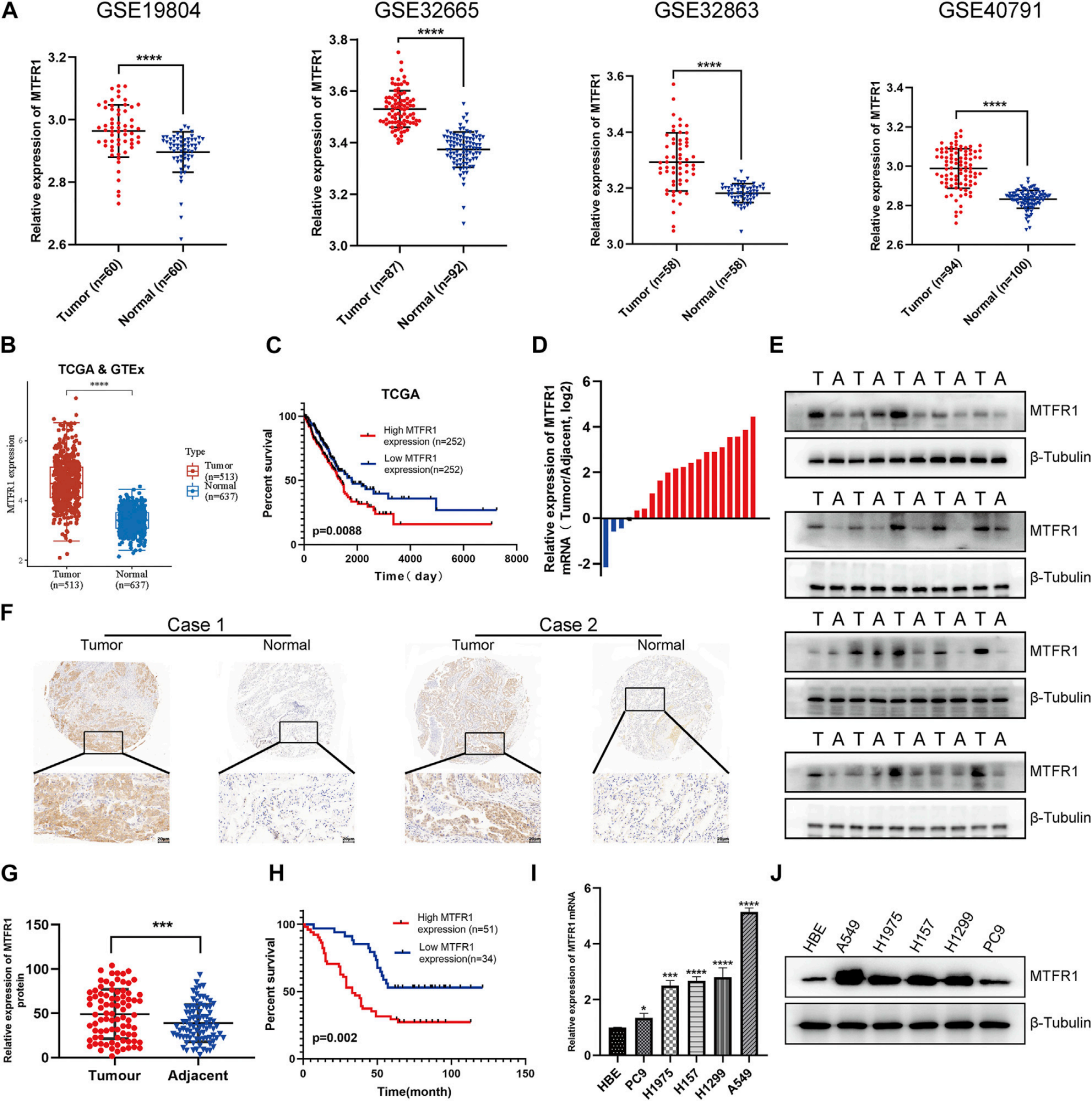
Mitochondrial fission regulator 1 (MTFR1) was overexpressed in lung adenocarcinoma (LUAD) cell lines and tissues. **(A)** The expression levels of MTFR1 in four Gene Expression Omnibus (GEO) datasets. **(B)** MTFR1 expression levels in The Cancer Genome Atlas (TCGA) and Genome–Tissue Expression (GTEx) databases. **(C)** Kaplan–Meier (KM) analysis for TCGA-LUAD samples. **(D, E)** The relative mRNA and protein expression of MTFR1 in 20 pairs of LUAD tumour (T) tissues and their matching adjoining (A) normal tissues. **(F)** Representative immunohistochemical images of LUAD and adjacent nonmalignant specimens stained for analysing MTFR1 expression. **(G)** The immunohistochemical score of 85 pairs of LUAD tissues and adjoining paracancerous tissues (*p* < 0.001). **(H)** Kaplan–Meier survival analyses of 85 patients with LUAD. **(I, J)** The relative protein and mRNA expression of MTFR1 in different cell lines. Data were articulated as the mean ± standard deviation (SD; *p < 0.05, ***p < 0.001, ****p < 0.0001).

First, we compared *MTFR1* gene expression between normal lung tissues and LUAD tissues using high-throughput data from the GEO, TCGA and GTEx databases. *MTFR1* was considerably overexpressed in LUAD tissues as compared with normal lung tissues (*p* < 0.0001, Figures 1A,B). The KM analysis revealed that elevated MTFR1 expression was associated with a poor prognosis in patients with LUAD in the TCGA cohort (*p* = 0.0088, Figure 1C). Furthermore, the mRNA and protein expression of MTFR1 was evaluated in 20 pairs of clinical samples. As demonstrated in Figures 1D,E, compared with those in the adjacent normal lung tissues, the protein and mRNA levels of MTFR1 were considerably elevated in LUAD tumour tissues. A total of 85 pairs of LUAD tissues and their corresponding adjoining normal lung tissues were subjected to IHC analysis with MTFR1 antibody, and the representative pictures are shown in Figure 1F. Furthermore, the expression of MTFR1 was quantified in each tissue sample. The result of IHC scoring revealed that the expression of MTFR1 was considerably elevated in LUAD tissues as compared with that in adjoining normal lung tissues. (*p* < 0.001, Figure 1G). The correlation between MTFR1 expression and clinical features of patients with LUAD was further examined. High MTFR1 expression was significantly related to the clinical stage (*p* = 0.007), lymph node metastasis (*p* = 0.016) and tumour size (*p* = 0.023) (Table 1). In addition, survival analysis revealed that patients with an elevated MTFR1 expression level exhibited worse clinical outcomes (*p* = 0.002, Figure 1H). Lastly, MTFR1 expression was examined in various cell lines. At both protein and mRNA levels, the expression of MTFR1 in LUAD cell lines was considerably higher than that in HBE cells (Figures 1I,IJ). Altogether, the results indicated that MTFR1 was upregulated in LUAD cells and tissues and associated with a poor prognosis in patients with LUAD.

**TABLE 1:** Correlation between MTFR1 expression and clinicopathological features in LUAD.

Clinicopathological factors	Sample	MTFR1 expression		*p* value
		High (H-score>40)	Low (H-score ≤40)	
Gender				
Male	50	29	21	0.822
Female	35	22	13	
Age(y)				
≥60	45	25	20	0.506
<60	40	26	14	
Smoking history				
Smoker	52	28	24	0.177
Nonsmoker	33	23	10	
Tumor size (cm)				
<3	21	8	13	*0.023
≥3	64	43	21	
Lymph node metastasis				
With	44	32	12	*0.016
Without	41	19	22	
Clinical stage				
Ⅰ ∼ Ⅱ	49	23	26	**0.007
Ⅲ ∼ Ⅳ	36	28	8	

LUAD, lung adenocarcinoma; ^*^ *p* < 0.05, ^**^ *p* < 0.01, χ2 test. High MTFR1 expression was significantly related to the clinical stage, lymphnode metastasis and tumour size.

### MTFR1 Promoted the Proliferation and Inhibited the Apoptosis of LUAD Cells

**FIGURE 2:**
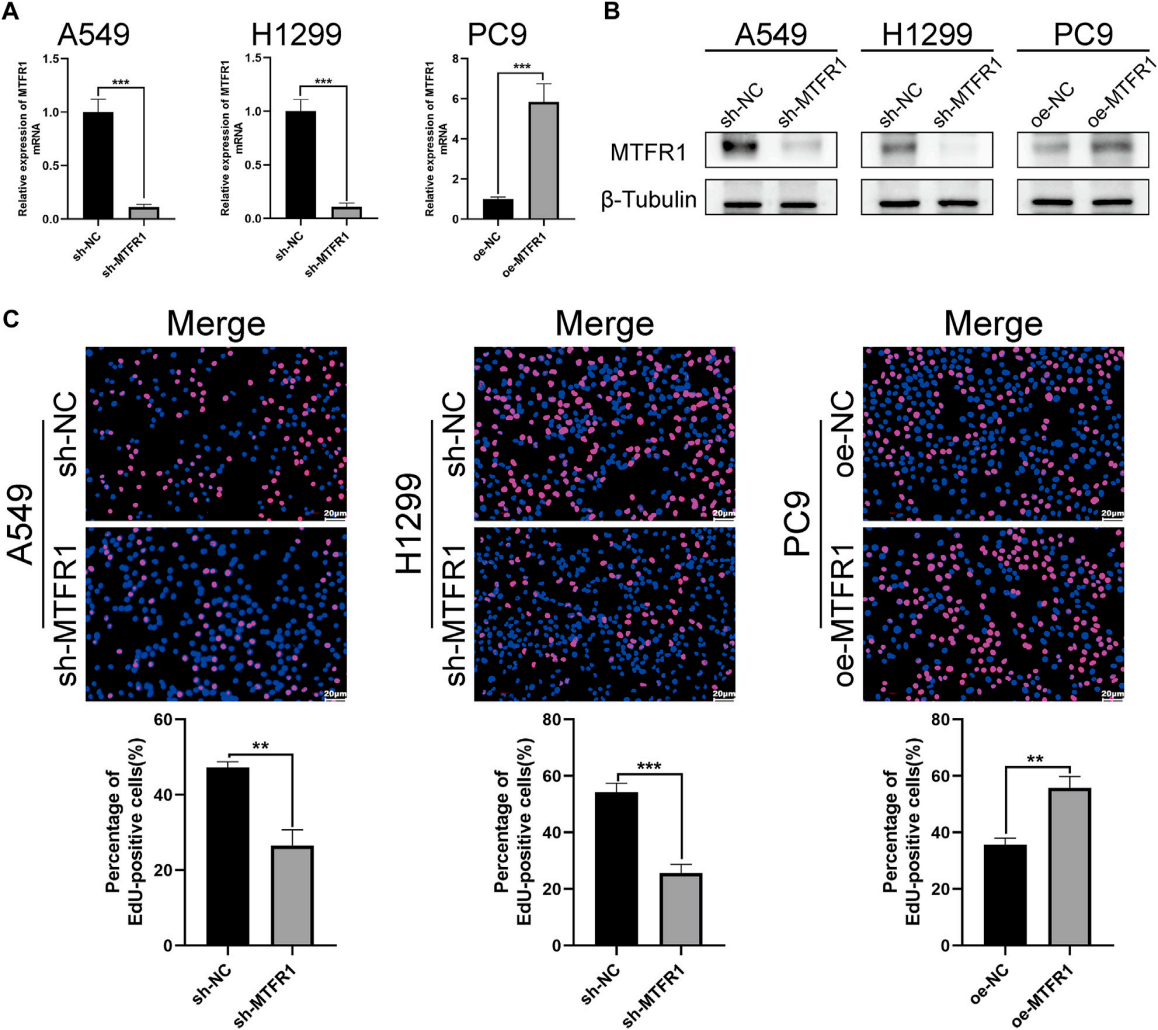
MTFR1 stimulated the proliferation of LUAD cells. **(A, B)** Evaluation of the effectiveness of lentiviral transduction by real-time quantitative polymerase chain reaction (RT-qPCR) and western blotting (WB). **(C)** EdU staining of the transduced cells. Data were articulated as the mean ± standard deviation (SD; **p < 0.01, ***p < 0.001).

**FIGURE 3:**
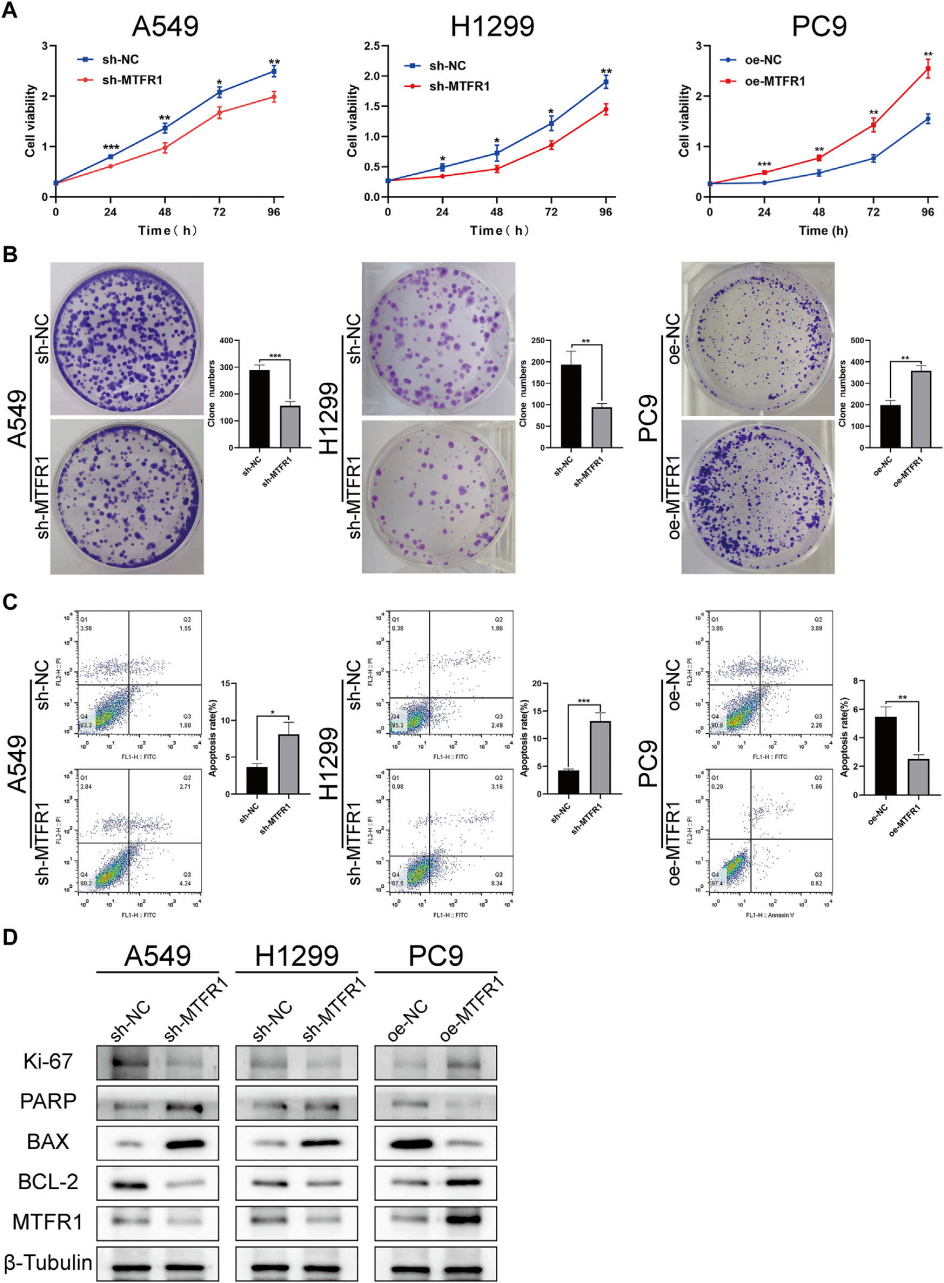
MTFR1 stimulated proliferation and suppressed apoptosis of LUAD cells. **(A)** Cell Counting Kit-8 (CCK-8) assay of the transduced cells. **(B)** Colony formation assay of the transduced cells. **(C)** Evaluation of apoptosis by flow cytometry. **(D)** Evaluation of the expression of cell proliferation and apoptosis-related protein by WB. Data were articulated as the mean ± standard deviation (SD; *p < 0.05, **p < 0.01 ***p < 0.001).

sh-RNA 2 had the best knockdown efficiency among these three sh-RNAs (Supplementary Table S1; Supplementary Figure S1A), so that it was selected for the subsequent experiments. Because MTFR1 was expressed at high levels in A549 and H1299 cells but at low levels in PC9 cells (Figures 1I,J). Therefore, we downregulated the expression of MTFR1 in A549 and H1299 cells and upregulated its expression in PC9 cells using lentiviral transduction. After transduction, MTFR1 expression was considerably downregulated in A549 and H1299 cells and upregulated in PC9 cells (Figures 2A,B). The proliferative ability of the stably transduced A549, H1299, and PC9 cells were then detected using EdU, colony formation and CCK-8 assays. The results of these assays revealed that the proliferative ability of sh-MTFR1 A549 and H1299 cells was significantly reduced as compared with that of the cells in the sh-NC groups. However, the proliferative ability of oe-MTFR1 PC9 cells was higher than that of PC9 cells in the oe-NC group (Figures 2C, 3A,B). Furthermore, flow cytometry was employed to identify the effects of MTFR1 on the cell cycle progression and apoptosis of LUAD cells. The results revealed that MTFR1 knockdown could promote cell apoptosis in A549 and H1299 cells, whereas MTFR1 upregulation could reduce apoptosis in PC9 cells (Figure 3C). In addition, we observed that MTFR1 had no apparent effect on cell cycle progression (figure not shown). Lastly, cell proliferation and apoptosis-related proteins were identified in transduced A549, H1299 and PC9 cells. The expression of Ki-67 and Bcl-2 proteins was decreased, whereas that of PARP and BAX proteins was increased after the knockdown of MTFR1 in H1299 and A549 cells. However, we observed opposite results after upregulating MTFR1 expression in PC9 cells (Figure 3D). These results indicated that MTFR1 promoted the proliferation and inhibited the apoptosis of LUAD cells.

### MTFR1 Promoted the Migration and Invasion of LUAD Cells

**FIGURE 4:**
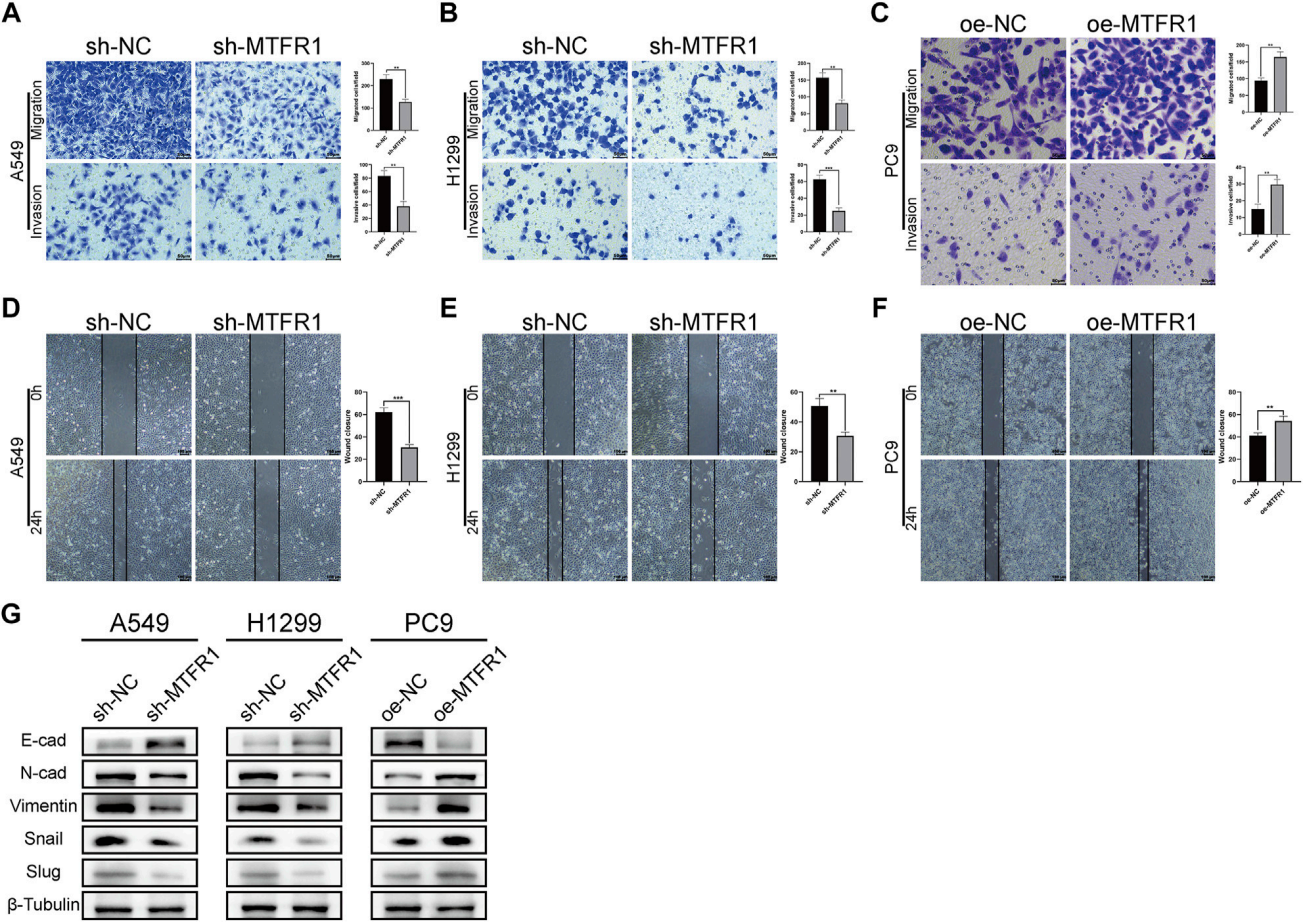
**(A–F)** Evaluation of the invasion and migration abilities of transduced cells by Transwell and wound-healing assays. **(G)** Evaluation of the levels of epithelial–mesenchymal transition (EMT)-related proteins by WB. Data were articulated as the mean ± standard deviation (SD; **p < 0.01 ***p < 0.001).

To assess the impact of MTFR1 on the migratory and invasive abilities of LUAD cancer cells, we performed Transwell and wound-healing assays. The results of the Transwell assay revealed that the numbers of invaded and migrated cells were considerably decreased after MTFR1 knockdown in H1299 and A549 cells (Figures 4A,B). Consistently, the numbers of invaded and migrated cells were considerably higher in the oe-MTFR1 cohort than in the oe-NC cohort (Figure 4C; *p* < 0.01). The results of the wound-healing assay revealed that MTFR1 downregulation in H1299 and A549 cells was associated with a considerably smaller wound closure area in the sh-MTFR1 cohort than in the NC cohort (Figures 4D,E; *p* < 0.001). However, when MTFR1 was upregulated in PC9 cells, the wound closure area was considerably larger in the oe-MTFR1 cohort than in the oe-NC cohort (Figure 4F; *p* < 0.01). Because epithelial–mesenchymal transition (EMT) is vital for tumour invasion and metastasis, we assessed the expression of EMT-related proteins in the transduced cells. The results revealed that MTFR1 knockdown decreased the expression of vimentin, N-cadherin, snail, and slug proteins but increased the expression of E-cadherin in H1299 and A549 cells. However, contradictory results were obtained when MTFR1 was upregulated in PC9 cells (Figure 4G). Altogether, these results suggested that MTFR1 stimulated the migration and invasion of LUAD cells.

### MTFR1 Regulated Glycolysis and the AMPK/mTOR Signalling Pathway in LUAD Cells

**FIGURE 5:**
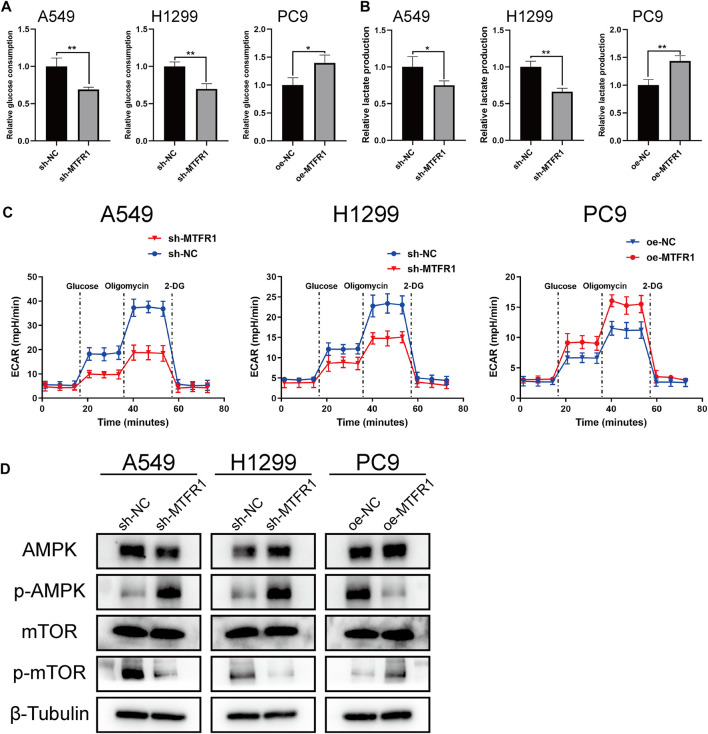
MTFR1 regulated glycolysis and AMPK/mTOR signalling pathway in LUAD cells. **(A)** Relative glucose consumption of the transduced cells. **(B)** Relative lactate production of the transduced cells. **(C)** Relative extracellular acidification rate (ECAR) of the transduced cells. **(D)** Evaluation of the expression of AMPK, p-AMPK, mTOR, and p-mTOR in the transduced cells by WB. Data were articulated as the mean ± standard deviation (SD; *p < 0.05, **p < 0.01).

To investigate whether MTFR1 affected glycolysis in LUAD cells, we measured the ECAR, the glucose consumption and lactate production rates in the transduced LUAD cells. The rates were increased in PC9 cells after MTFR1 overexpression whereas decreased in H1299 and A549 cells after MTFR1 downregulation (Figures 5A–C). These findings indicated that MTFR1 promoted aerobic glycolysis in LUAD cells.

Many studies have reported that the AMPK/mTOR signalling pathway is involved in the regulation of tumour metabolism. Therefore, we detected the expression of the major molecules that participate in the AMPK/mTOR pathway (mTOR, p-mTOR, AMPK, and p-AMPK). The results indicated that the expression of p-MTOR was reduced whereas that of p-AMPK was elevated when MTFR1 expression was downregulated in H1299 and A549 cells. However, contrasting effects were obtained when MTFR1 expression was upregulated in PC9 cells (Figure 5D). In conclusion, MTFR1 may exert its pro-oncogenic functions by targeting the AMPK/mTOR signalling pathway in LUAD cells.

### MTFR1 was Directly Targeted by miR-29c-3p

**FIGURE 6:**
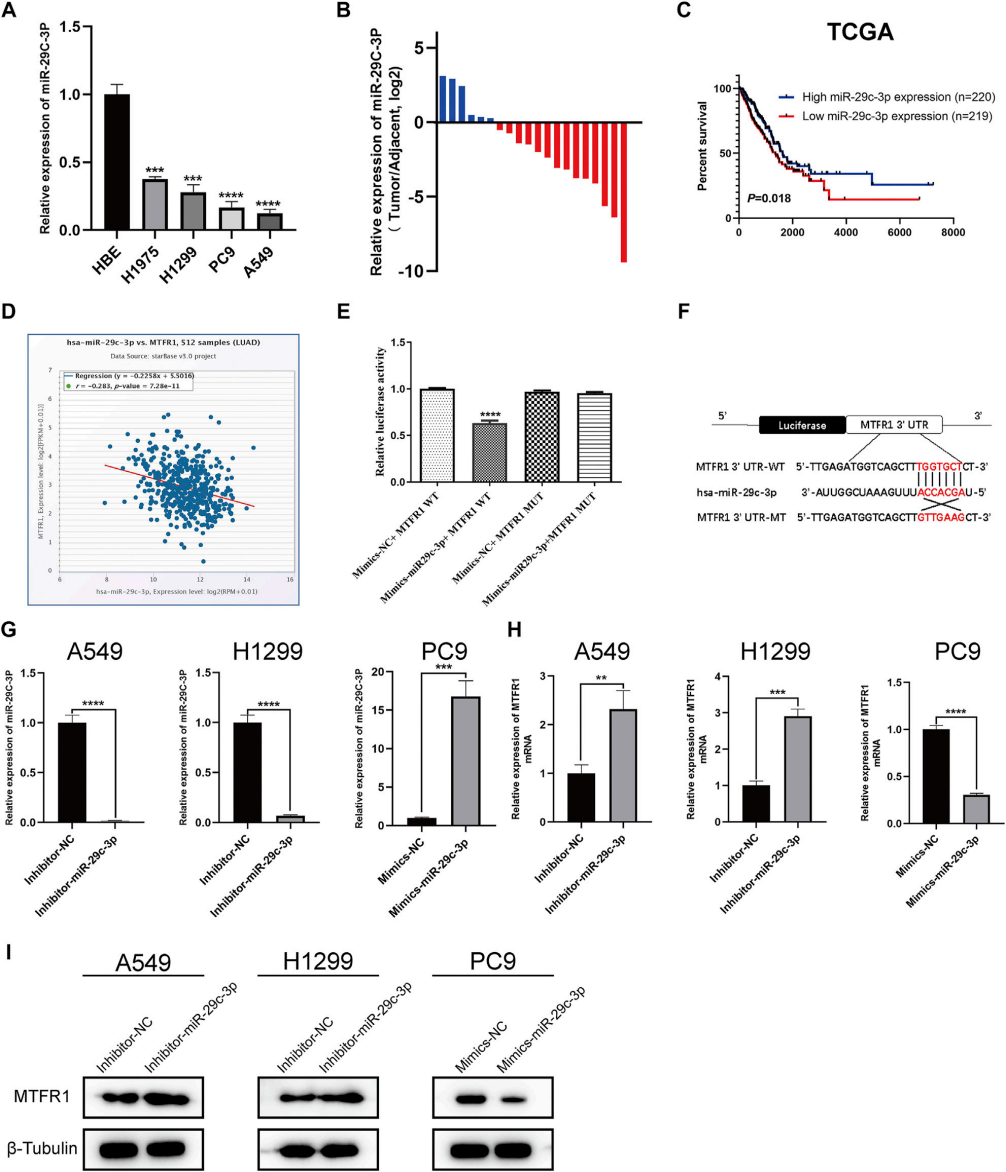
MTFR1 is directly targeted by miR-29c-3p. **(A)** miR-29c-3p expression in different cell lines. **(B)** The relative expression of miR-29c-3p in 20 pairs of LUAD tissues and their corresponding adjoining paracancerous tissues. **(C)** Kaplan–Meier analysis of TCGA LUAD samples based on miR-29c-3p expression. **(D)** Pearson’s correlation analysis for assessing the relationship between miR-29c-3p and MTFR1 expression. **(E, F)** Results of the dual-luciferase reporter assay and the predicted binding site between miR-29c-3p and MTFR1 3′-UTR. **(G–I)** The expression of miR-29c-3p and MTFR1 in cells transfected with inhibitor-miR-29c-3p or mimic-miR-29c-3p. Data were articulated as the mean ± standard deviation (SD; **p < 0.01, ***p < 0.001, ****p < 0.0001).

First, we predicted the upstream miRNAs of MTFR1 based on bioinformatic analysis and chose three of them for primary study (Supplementary Tables S2, S3). After transfecting the inhibitors and mimics of these three miRNAs into the corresponding cells, we observed that miR-29c-3p had the most obvious effects on MTFR1 expression (Supplementary Figure S1B–D). Thus, we eventually selected miR-29c-3p for further study. At the cellular level, miR-29c-3p expression was noticeably higher in HBE cells than in the four LUAD cell lines (Figure 6A). At the tissue level, miR-29c-3p expression was higher in paracancerous tissues than that in the LUAD tissues (Figure 6B). Moreover, KM survival analysis demonstrated that elevated expression of miR-29c-3p was associated with a good prognosis in patients with LUAD in the TCGA cohort (*p* = 0.018; Figure 6C). Furthermore, we searched the starBase database to assess the relationship between MTFR1 and miR-29c-3p expression in patients with LUAD. We found miR-29c-3p expression exhibited an inverse relationship with MTFR1 expression (Figure 6D; r = −0.283, *p* < 0.0001). Subsequently, we performed the dual-luciferase reporter assay to further assess whether a direct regulatory correlation existed between MTFR1 and miR-29c-3p. Fluorescence intensity in the mimics-miR-29c-3p + MTFR1 WT cohort was lower than that in the other cohorts (*p* < 0.0001) as demonstrated in Figure 6E. These results indicated that miR-29c-3p could directly bind to the 3′-UTR of MTFR1 mRNA. Figure 6F demonstrates the predicted binding sites between *MTFR1* mRNA and miR-29c-3p. Lastly, we transfected A549 and H1299 cells with inhibitor-miR-29c-3p and PC9 cells with mimics-miR-29c-3p to evaluate the expression of miR-29c-3p and MTFR1. We observed that when miR-29c-3p expression was upregulated in H1299 and A549 cells, MTFR1 was downregulated at both protein and mRNA levels; however, when miR-29c-3p expression was downregulated in PC9 cells, MTFRI was upregulated at both protein and mRNA levels (Figures 6G–I). In conclusion, these results revealed that MTFR1 was directly targeted by miR-29c-3p.

### miR-29c-3p Could Partly Rescue the Phenotypic Changes Caused by MTFR1

**FIGURE 7:**
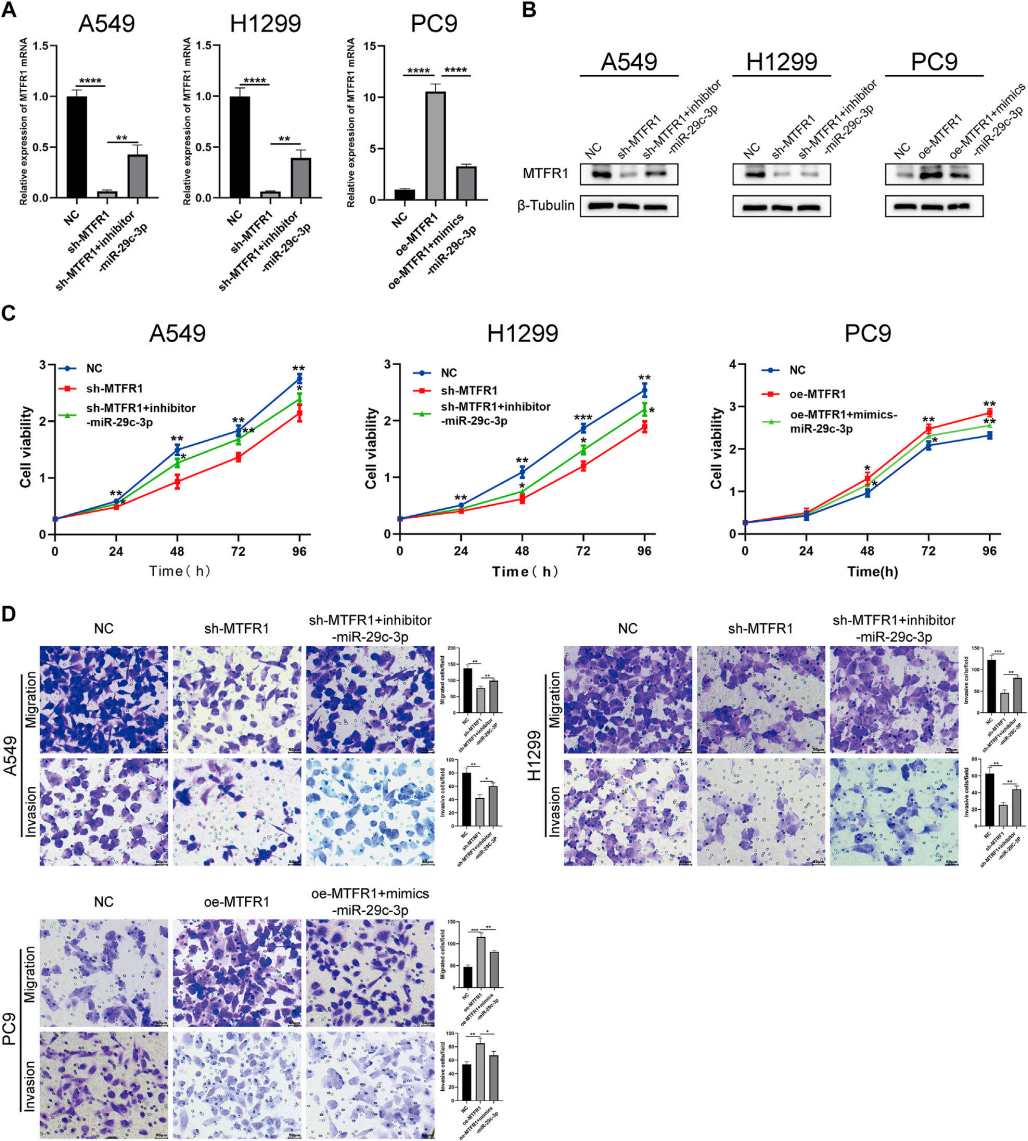
miR-29c-3p could partly rescue the phenotypic changes caused by MTFR1. **(A, B)** Transfection efficacy validated using real-time quantitative polymerase chain reaction (RT-qPCR) and WB. **(C)** miR-29c-3p could rescue the effect of MTFR1 on the proliferation of LUAD cells. **(D)** miR-29c-3p could rescue the effect of MTFR1 on the invasion and migration of LUAD cells. Data were articulated as the mean ± standard deviation (SD; *p < 0.05, **p < 0.01, ***p < 0.001, ****p < 0.0001).

**FIGURE 8:**
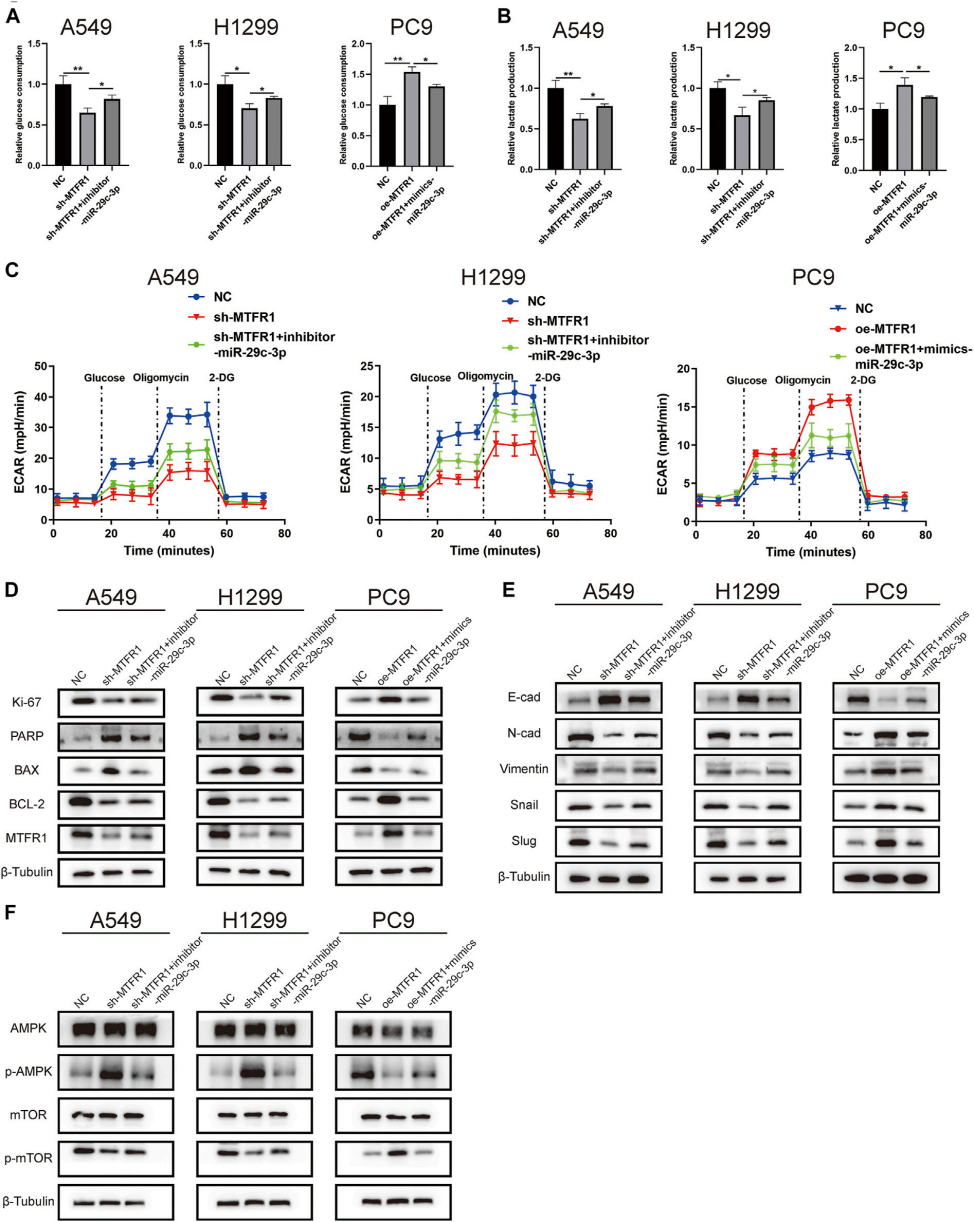
miR-29c-3p could partly rescue the changes of glycolytic capacity and the expression of the phenotype-associated proteins caused by MTFR1 in LUAD cells. **(A–C)** miR-29c-3p could rescue the effect of MTFR1 on the glycolytic capacity of LUAD cells. **(D–F)** miR-29c-3p could reverse MTFR1-induced alterations in the expression of proteins associated with cell proliferation, apoptosis, EMT and the AMPK/mTOR signalling pathway in LUAD cells. Data were articulated as the mean ± standard deviation (SD; *p < 0.05, **p < 0.01).

We performed rescue experiments to assess whether MTFR1 was functionally regulated by miR-29c-3p. First, we transfected sh-MTFR1 A549 and H1299 cells with inhibitor-miR-29c-3p and oe-MTFR1 PC9 cells with mimics-miR-29c-3p and evaluated the transfection efficiency at both protein and mRNA levels (Figures 7A,B). Furthermore, the results of CCK-8 and Transwell assays revealed that miR-29c-3p could reverse the phenotypic changes on the proliferation, migration and invasion of LUAD cells caused by MTFR1 (Figures 7C,D). Moreover, the evaluation of ECAR, glucose and lactate levels indicated that miR-29c-3p could rescue the changes in glycolytic capacity induced by MTFR1 (Figures 8A–C). Moreover, the expression of proteins associated with cell proliferation, apoptosis, EMT and AMPK/mTOR pathway changed accordingly (Figures 8D–F). Altogether, the results indicated that phenotype changes induced by MTFR1 could be reversed by miR-29c-3p.

### MTFR1 Promoted Tumour Growth and Metastasis *in Vivo*

**FIGURE 9:**
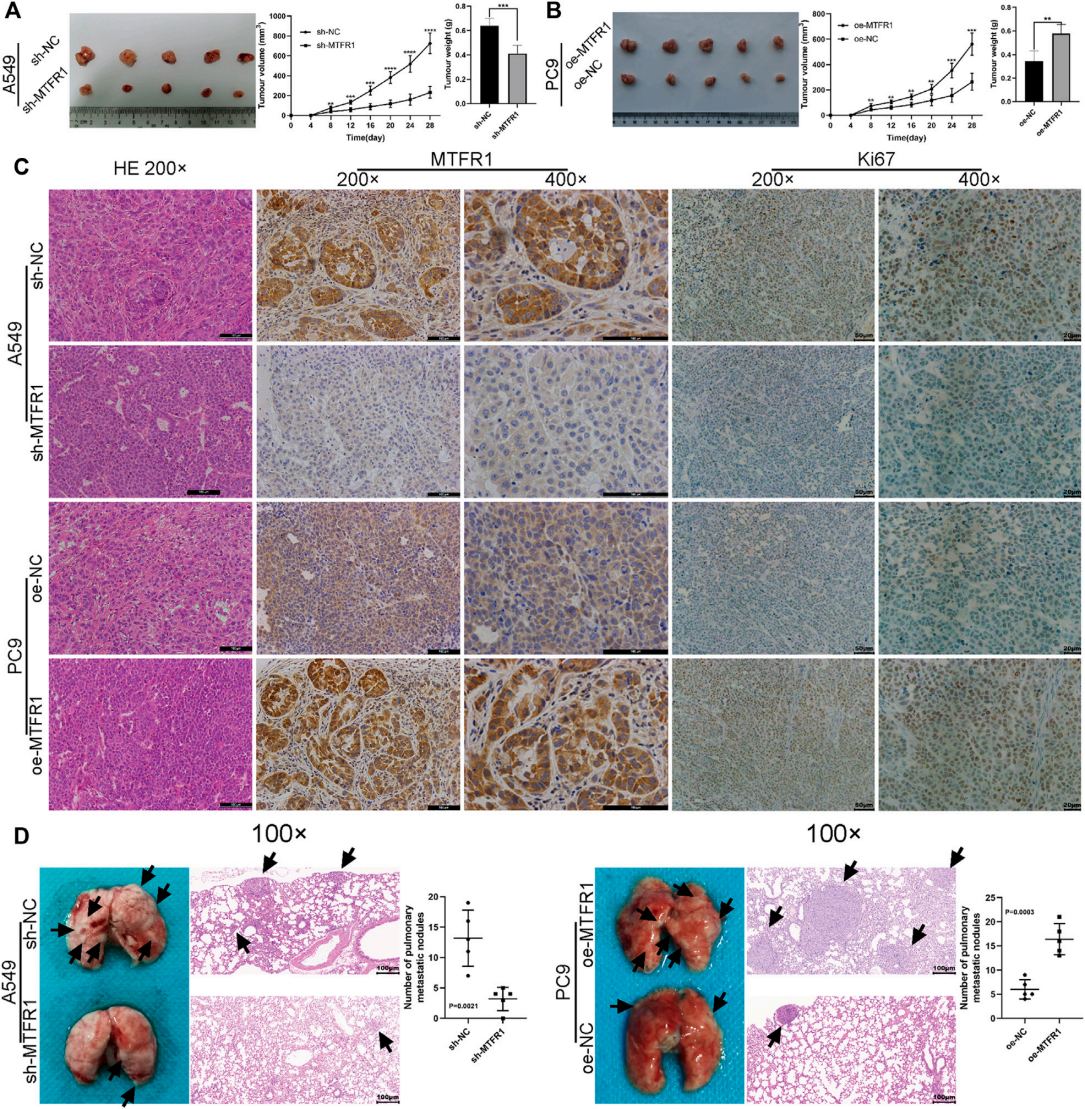
MTFR1 promoted the progression of LUAD cells *in vivo*. **(A,B)** Tumour images, tumour growth curves and tumour weights of each group. **(C)** Haematoxylin–eosin (HE) staining and immunohistochemical analysis of the Ki-67 and MTFR1 proteins of tumours in each group. **(D)** Photographs (left) and HE-stained images (right) of the lungs in each group; the black arrowheads denote lung metastasis nodules (**p < 0.01, ***p < 0.001, ****p < 0.0001).

A tumour xenograft-harbouring nude mouse model and a tail-vein injection-induced metastasis model were constructed to assess the *in vivo* impacts of MTFR1 on tumour growth and metastasis. Tumours in the sh-NC cohort exhibited a faster growth rate than that of the sh-MTFR1 cohort. Moreover, the average tumour weight and volume in the sh-NC cohort were significantly higher than those in the sh-MTFR1 cohort (Figure 9A). Simultaneously, tumours in the oe-MTFR1 cohort exhibited a faster growth rate and a significantly higher average weight and volume than those in the oe-NC group (Figure 9B). Furthermore, HE staining and IHC analysis were performed on tumour xenografts to analyse Ki-67 and MTFR1 expressions. Groups with high MTFR1 expression exhibited higher Ki-67 expression than that exhibited by groups with low MTFR1 expression (Figure 9C). The results of the lung metastasis model assay revealed that MTFR1 overexpression increased and MTFR1 knockdown decreased the number of lung metastatic nodules (Figure 9D). Altogether, the results indicated that MTFR1 promoted LUAD tumour growth and metastasis *in vivo*.

## Discussion

This study reported for the first time that MTFR1 was markedly overexpressed in LUAD cell lines and tissue samples. Moreover, high expression of MTFR1 was associated with adverse clinicopathological features and poor overall survival of LUAD patients. Findings from both *in vivo* and *in vitro* experiments demonstrated that overexpressed MTFR1 stimulated the proliferation, invasion, migration and glycolytic capacity and repressed the apoptosis of LUAD cells, whereas MTFR1 knockdown exerted contradictory effects. In addition, we discovered that miR-29c-3p was a direct upstream target of MTFR1 and could inhibit the cancer-promoting functions of MTFR1. Furthermore, we also discovered that MTFR1 exerts its biological functions by regulating the AMPK/mTOR signalling pathway. All these results indicate that MTFR1 suppression may serve as a new therapeutic strategy for treating patients with LUAD.

In recent years, the essential role of mitochondrial dynamics (fusion/fission) in the development of cancers has gained increasing recognition. Rehman et al. reported that mitochondrial fission promotes the progression of lung cancer (Rehman et al., 2012). In addition, Zhao et al. suggested that mitochondrial fission also promotes the progression of breast cancer. MTFR1, a mitochondrial protein, facilitates mitochondrial fission. In addition, MTFR2, which belongs to the same family as MTFR1, promotes the progression of breast cancer and oral squamous carcinoma when its expression is dysregulated (Lu et al., 2019; Wang W. et al., 2020). However, to date, only a few studies have used bioinformatic analyses to assess whether MTFR1 is involved in the development of cancers (Wang et al., 2015; Reddy et al., 2019; Sanada et al., 2019). In our study, we demonstrated that MTFR1 was abnormally upregulated in patients with LUAD and might act as a prognostic marker. Moreover, MTFR1 promoted the progression of LUAD. Therefore, our study provided an ideal biomarker for the diagnosis, therapeutic intervention and prognosis of LUAD.

Metabolic reprogramming has been recognised as one of the most remarkable hallmarks of cancer cells. Metabolic reprogramming in cancer cells can be best explained by the Warburg effect, in which cancer cells preferentially use glycolysis for energy generation regardless of the presence of oxygen (Vander Heiden et al., 2009). As mentioned previously, cancer cells can regulate mitochondrial dynamics to meet their metabolic requirements. Therefore, we hypothesised that MTFR1 could regulate the Warburg effect by altering mitochondrial dynamics in LUAD cells. To verify our hypothesis, we analysed the ECAR, the glucose consumption and lactate production rates of the transduced cells. We found that when MTFR1 expression was upregulated, the ECAR and the rates of glucose consumption and lactate production in LUAD cells were concomitantly elevated. However, the opposite result was observed when MTFR1 expression was downregulated. These findings indicate that MTFR1 stimulates the Warburg effect of LUAD cells. Our findings offer a new understanding of the treatment of LUAD by targeting cancer metabolism.

The occurrence and development of LUAD are accompanied by the alteration of multiple signalling pathways. Among these pathways, the AMPK/mTOR pathway is closely associated with tumour metabolism (Han et al., 2013). AMPK is considered an energy status sensor that contributes to maintaining cellular energy homeostasis. In addition, AMPK performs other functions such as regulation of cell growth and proliferation, autophagy, mitochondrial biogenesis and recycling and maintenance of cell polarity (Hardie, 2011). mTOR, which is negatively regulated by AMPK, is a central integrator of both growth factors and nutrient signals. It regulates numerous cellular processes including cell growth, cell cycle and angiogenesis (Chapuis et al., 2010). Previous studies have indicated that targeting AMPK/mTOR may be an efficient strategy for treating NSCLC (Han et al., 2013). Based on these studies and our finding that MTFR1 could promote aerobic glycolysis in LUAD, we hypothesised that MTFR1 could perform its biological function by regulating the AMPK/mTOR signalling pathway. Therefore, we identified the expression levels of several critical proteins of the AMPK/mTOR signalling pathway. As expected, MTFR1 overexpression elevated p-AMPK expression and reduced p-mTOR expression. Opposing findings were observed when MTFR1 expression was downregulated. These results confirmed our hypothesis that MTFR1 expression changes could change the expression of AMPK/mTOR signalling pathway and it may perform its biological through this pathway.

miRNAs are a class of non-coding RNAs that have been extensively studied, and aberrant miRNA expression is associated with many human diseases, including various cancers (Jiang et al., 2009). In our study, we predicted and validated miR-29c-3p as a direct upstream miRNA of MTFR1. miR-29c-3p expression was not only downregulated in LUAD cells but also inversely correlated with MTFR1. Furthermore, we performed rescue experiments to confirm that miR-29c-3p might partly rescue the malignant phenotypes induced by MTFR1. These findings indicate that the miR-29c-3p/MTFR1/AMPK/mTOR axis plays an important role in LUAD. However, because MTFR1 exerts robust cancer-promoting effects on LUAD, further investigation is required to identify proteins that interact with MTFR1 to exercise such effects.

In conclusion, MTFR1 serves as a strong tumour-promoting factor in LUAD, and targeting MTFR1 may provide a better disease outcome for patients with LUAD.

## Data Availability

The original contributions presented in the study are included in the article/Supplementary Material, further inquiries can be directed to the corresponding author.
