# Corrigendum: Negatively regulated by miR-29c-3p, MTFR1 promotes the progression and glycolysis in lung adenocarcinoma via the AMPK/mTOR signalling pathway

**DOI:** 10.3389/fcell.2022.870313

**Published:** 2022-08-31

**Authors:** Yongmeng Li, Yanfei Liu, Kai Jin, Rui Dong, Cun Gao, Libo Si, Zitong Feng, Huiying Zhang, Hui Tian

**Affiliations:** ^1^ Department of Thoracic Surgery, Qilu Hospital, Cheeloo College of Medicine, Shandong University, Jinan, China; ^2^ Department of Anesthesiology, Qilu Children’s Hospital of Shandong University, Jinan, China

**Keywords:** lung cancer, microRNA, mitochondrial fission regulator 1, warburg effect, proliferation, invasion, migration

In the original article, there was a mistake in Figure 9D as published. When we were arranging the panel, we misused the HE staining pictures by using the wrong folder. The corrected Figure 9D appears below.

**FIGURE 9:**
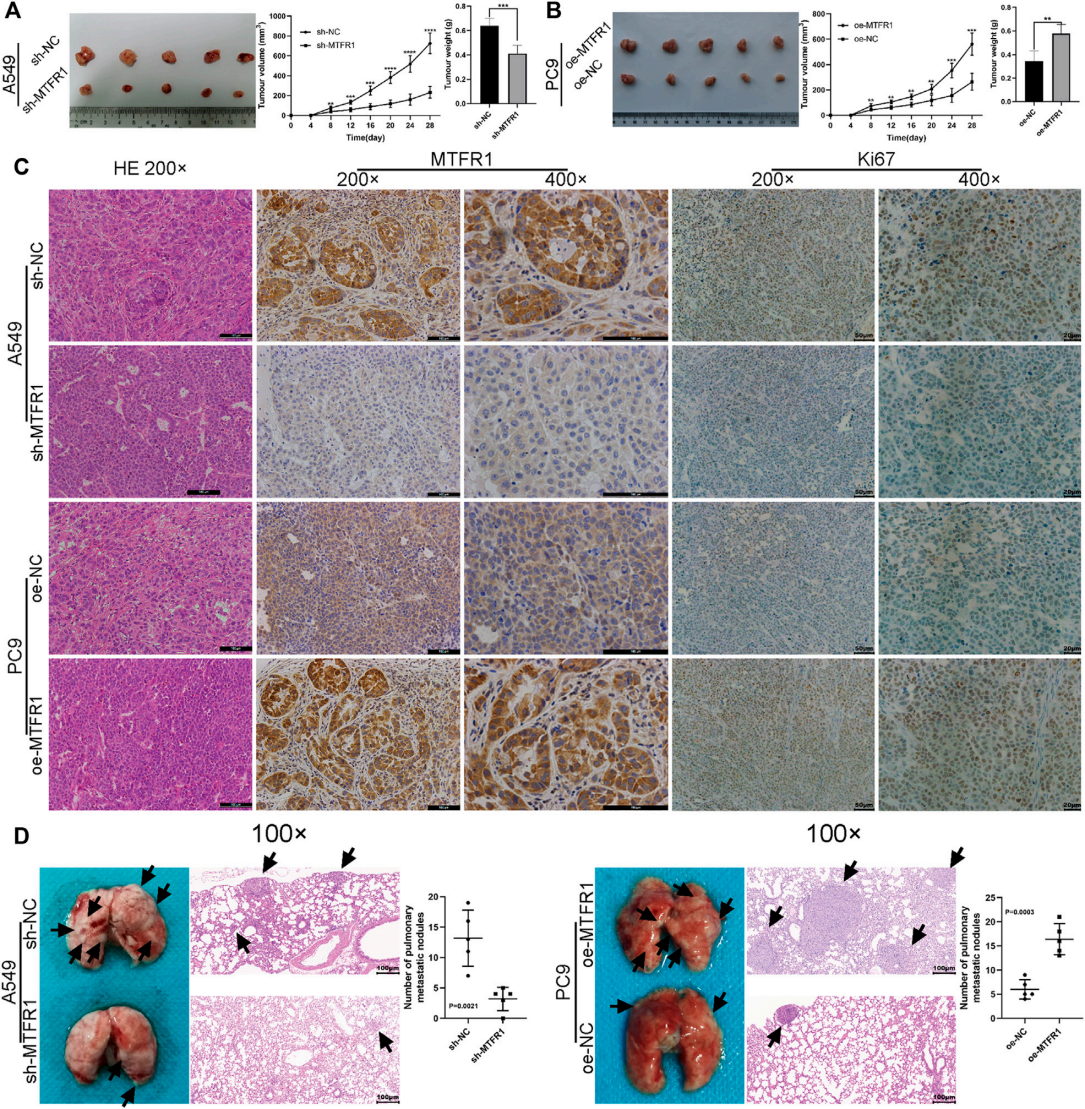
MTFR1 promoted the progression of LUAD cells *in vivo*. **(A,B)** Tumour images, tumour growth curves and tumour weights of each group. **(C)** Haematoxylin–eosin (HE) staining and immunohistochemical analysis of the Ki-67 and MTFR1 proteins of tumours in each group. **(D)** Photographs (left) and HE-stained images (right) of the lungs in each group; the black arrowheads denote lung metastasis nodules (**p < 0.01, ***p < 0.001, ****p < 0.0001).

The authors apologize for this error and state that this does not change the scientific conclusions of the article in any way. The original article has been updated.

