# Supplementary figures and images for: Negatively Regulated by miR-29c-3p, MTFR1 Promotes the Progression and Glycolysis in Lung Adenocarcinoma via the AMPK/mTOR Signalling Pathway
Source: Front Cell Dev Biol. 2021 Dec 1;9:771824. doi: 10.3389/fcell.2021.771824 (PMC8672271; doi:10.3389/fcell.2021.771824)

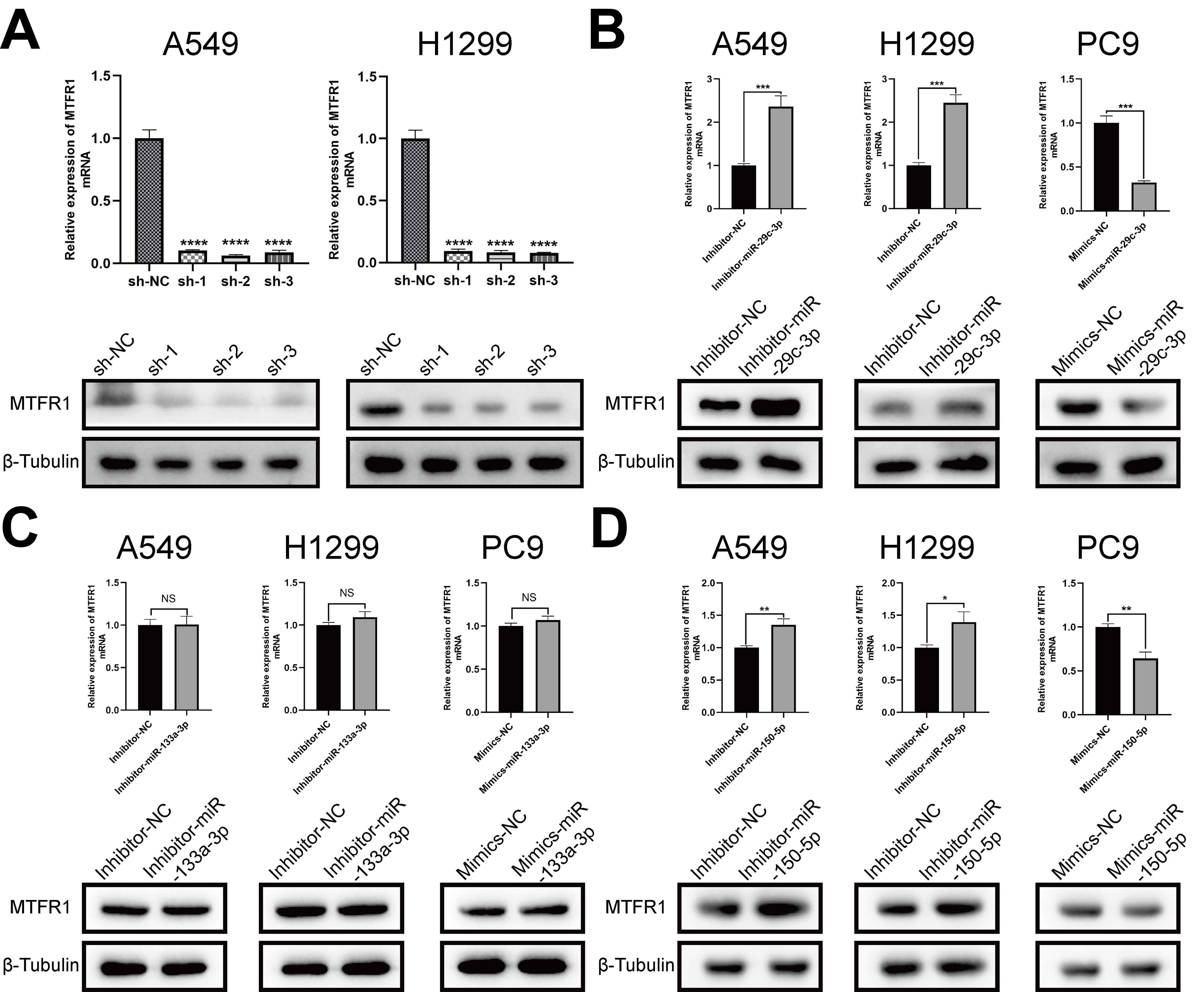

Supplement: Supplementary file 1 [file Image1.TIF]
