# Supplementary material for: Negatively Regulated by miR-29c-3p, MTFR1 Promotes the Progression and Glycolysis in Lung Adenocarcinoma via the AMPK/mTOR Signalling Pathway
Source: Front Cell Dev Biol. 2021 Dec 1;9:771824. doi: 10.3389/fcell.2021.771824 (PMC8672271; doi:10.3389/fcell.2021.771824)
Supplement: Supplementary file 2 [file DataSheet1.PDF]

Legends as for Supplementary Figure 1:

(A) Interference efficiency of the three sh-MTFR1 lentivirus in A549 and H1299 cells. (B-D) MTFR1 expression was measured by real-time quantitative polymerase chain reaction (RT-qPCR) and western blotting (WB) after transfecting the inhibitors and mimics of the three miRNAs into the corresponding cells. Data were articulated as the mean ± standard deviation (SD; *P <0.05, ** P <0.01, *** P <0.001, **** P <0.0001).

Supplementary Table 1. The sequences of sh-NC and three sh-RNAs of MTFR1

Name	Sequence（5′-3′）
sh-MTFR1-NC	5′-TTCTCCGAACGTGTCACGT-3′
sh-MTFR1-1	5′-GCTGGATTAAGCGCCTAAT-3′
sh-MTFR1-2	5′-GCAGGTGACTTAGATTCTA-3′
sh-MTFR1-3	5′-GCTGGAAAGACTTTGGTTA-3′

Supplementary Table 2. The primer sequences of miR-29c-3p, 133a-3p and miR-150-5p

Name		Sequence（5′-3′）
miR-29c-3p	Forward	5′-CTCCTCCTTTTAGCACCATTTG-3′
	Reverse	5′-TATGCTTGTTCTCGTCTCTGTGTC-3′
miR-133a-3p	Forward	5′-GCCTTTGGTCCCCTTCAAC-3′
	Reverse	5′-TATGCTTGTTCTCGTCTCTGTGTC-3′
miR-150-5p	Forward	5′-CAGTATTCTCTCCCAACCCTTGTA-3′
	Reverse	5′-TATGGTTTTGACGACTGTGTGAT-3′

Supplementary Table 3. The sequences of the inhibitors and mimics of miR-29c-3p, 133a-3p and miR-150-5p

Name	Sequence（5′-3′）
Inhibitor-NC	5′-CAGUACUUUUGUGUAGUACAA-3′
Inhibitor-miR-29c-3p	5′-UAACCGAUUUCAAAUGGUGCUA-3′
Inhibitor-miR-133a-3p	5′-CAGCUGGUUGAAGGGGACCAAA-3′
Inhibitor-miR-150-5p	5′-CACUGGUACAAGGGUUGGGAGA-3′

Mimics-NC	5′-UUCUCCGAACGUGUCACGUTT-3′
Mimics-miR-29c-3p	5′-UAGCACCAUUUGAAAUCGGUUA-3′
Mimics-miR-133a-3p	5′-UUUGGUCCCCUUCAACCAGCUG-3′
Mimics-miR-150-5p	5′-UCUCCCAACCCUUGUACCAGUG-3′
